# Development and Supervision of Financial Technology Based on Blockchain

**DOI:** 10.1155/2022/2615153

**Published:** 2022-06-01

**Authors:** Rui Yang

**Affiliations:** College of Economic and Management, Chongqing Industry Polytechnic College, Chongqing 401120, China

## Abstract

Decentralization, stability, security, and immutability are all features of blockchain technology. Blockchain, as the underlying technology of Bitcoin's digital monetary system, is currently sweeping the globe. Blockchain is a revolutionary decentralized database technology that employs encryption, a timestamp chain data structure, a distributed consensus mechanism, and other technologies to achieve decentralization, tamper resistance, easy tracking, and programmable smart contracts. In the face of rising financial technology, we must maintain inclusive, technological, and invasive regulatory principles that not only foster financial innovation, but also conduct dynamic supervision to avoid systemic financial hazards. The consensus algorithm is one of the main blockchain technologies that has a direct impact on the system's functioning. As a result, in this paper, we propose a blockchain-based development and supervision method for financial technology, as well as an application of this technology to commercial settlement, which can significantly reduce data complexity, time consumption, and the structural chain phenomenon in existing transaction settlement. We bring the idea of pow competition into DPoS, construct a consensus algorithm with an upgrade mechanism, and call it delegated proof of work, based on an in-depth investigation of the working principle of pow (proof of work) (dDPoS). The blocking efficiency of the dDPoS consensus method is around one block every 10 seconds, which is significantly higher than the blocking efficiency of the POW and POS consensus algorithms. As a result, it offers a potential answer to traditional centralized institutions' concerns of high brokerage costs and insecure central storage, as well as a wide range of application possibilities.

## 1. Introduction

Financial English “Fin Tech” is an abbreviation for “Financial Technology,” which refers to the integration of finance and IT, as well as the use of various scientific and technological techniques to improve efficiency and successfully cut operating costs in the traditional financial industry [1]. Financial liberalization and globalization, on the other hand, make the entire financial system more vulnerable. Financial supervision has become a focal point of concern, particularly during the 1990s, as a result of massive, devastating, and recurring financial crises [2]. The price of Bitcoin has witnessed two strong surges and falls in recent years, owing to the progressive popularization of the notion of Bitcoin and the influence of investor mood and government supervision, which has sparked broad discussion and interest [3]. Although technology is transforming China's financial industry, it is also posing new challenges to legal concepts, norms, and regulatory structures [4]. The financial legal system is experiencing new issues as a result of decentralization, as represented by blockchain, and legal supervision reform, as represented by supervision technology [5].

The legislation of financial supervision and the financial industry is complementary. The financial industry's growth encourages financial supervision law innovation, and the financial supervision legislation ensures the financial industry's healthy growth [6]. On the other hand, if the legal structure of financial supervision cannot keep up with the financial industry's expansion, the financial business outside of supervision may provide unanticipated hazards [7]. Blockchain has a strong basis of trust as a sharing technology [8]. However, the blockchain technology development cycle is short, and the technology's evaluation is mixed [9]. The existing calculating methods may lead to a complex conversion procedure and low efficiency of long-term encryption and decryption process [10], yet all nodes in the blockchain network maintain the same block sequence and attain agreement according to the accepted norms [11]. In this research, we present the dDPoS algorithm and compare the experience with existing methods to verify the performance of the proposed method. This study examines the development and oversight of blockchain technology in financial settlements, as well as the associated concepts of blockchain technology, and explains, summarizes, and summaries them.

People began to recognize the usefulness of blockchain technology as the basic underlying technology when the price of bitcoin fluctuated dramatically [12]. Although blockchain is undeniably appealing to the banking industry, most banks are still in the early stages of adopting it, and many financial businesses are currently proving the concept and developing their own blockchain strategies or are only now becoming aware of it [13]. Furthermore, financial technology has altered the manner in which the financial industry operates, rather than the nature of the industry [14, 15]. Financial regulatory authorities in industrialized nations have been exploring how to monitor complex financial institutions, which has become the most significant responsibility, in order to achieve financial stability and avert financial crises. We should be aware of new hazards as a result of financial technology, not just the changes in the financial business. The efficient and complete evaluation of consensus algorithm is realized in this paper's financial technology development and supervision method based on blockchain, in order to discuss its working principle on private blockchain platform and how it affects the overall performance of blockchain, as well as the efficient and complete consensus algorithm, complete analysis and comparison of performance characteristics of various consensus algorithms, and a complete evaluation of consensus algorithms.

1. This study analyzes and describes the technological model, advantages and disadvantages of blockchain technology in as much detail as possible, based on a complete analysis of the existing academic literature, various research reports, and white papers in the industry.
2. The selected nodes can be the initial permanent nodes with resources and the right to vote in the DPoS process, and the PoW is used to “screen” the node sets with a specific computational capacity to vote in the election.
3. The use of blockchain technology in financial technology development and supervision can improve the loan quality and procedure in the financial industry, as well as ensuring loan business efficiency and endorsement transparency.

This paper's research framework is divided into five sections, which are as follows:

The first section of this paper introduces the research background and significance before moving on to the paper's primary work. The second section introduces the work on financial technology development and oversight, as well as blockchain and related algorithms. The third section analyzes the combination of financial efficiency and supervision, as well as structural optimization methodologies, to provide readers with a better knowledge of visual composition and semantic expression. The fourth section is the meat of the study, and it covers two areas of blockchain technology: blockchain core technology and consensus algorithm analysis. The final section of the paper is a recap of the entire study's work.

## 2. Related Work

### 2.1. Financial Technology Development and Supervision

Financial technology refers to financial innovations that create new business models, applications, processes, or products through technology. These innovations can have a significant impact on financial markets, financial institutions, or financial service delivery methods. Although deregulation and self-regulation can maintain market competitiveness, it is difficult to cope with the actual financial crisis, and international arbitrage has increased due to deregulation. In the face of the crisis, it has become an important topic for Chinese and foreign legal circles to have some thoughts, opinions, and constructive opinions, and how to improve the current financial supervision mode.

Zachariadis and others believe that regulatory adjustment is necessary because there are external influences that lead to serious information asymmetry, natural monopoly, and market failure in the financial market [16]. Kabra et al. analyzed the mechanism and application of blockchain consensus algorithm in the field of financial supervision, chose Java Spring framework as the implementation framework of the platform, and adopted Dockers technology to realize multi-node private chain deployment [17]. Cretarola and others systematically studied the supervision mode of world commercial banks and its relationship with financial development. However, the effectiveness of various financial supervision theories has not been systematically verified in the existing literature [18]. Egelund Muller and others believe that the public interest theory lacks empirical support, and the reason why many industries are regulated is not because of the higher degree of monopoly or easier to trigger monopoly [19]. The public interest theory of financial supervision proposed by Yu et al. is based on three assumptions: sufficient government information, contribution to the overall welfare of society, and sufficient credit. It allows individuals to supervise powerful financial institutions [20].

The financial supervision law needs to determine the future financial supervision reform plan from the perspective of national historical development. In terms of respecting and understanding China's actual situation, this paper attempts to explore a practical legal system of financial supervision, so as to create a platform for others and themselves to continue their research in the future.

### 2.2. Blockchain and Its Related Algorithms

In order to meet the needs of social development, the blockchain is constantly changing, and the core technology of blockchain-consensus algorithm also needs to be innovated. For example, Ping An Yizhang chain solution, Wanxiang blockchain laboratory baas platform, Zhonghui interbank joint loan clearing project, ant financial service social welfare project, etc. all show that domestic financial technology enterprises attach importance to blockchain technology application research and consensus algorithm. Many scholars at home and abroad have done a lot of in-depth research on financial supervision and investigated in detail the impact of various specific financial supervision measures on financial development.

Sun et al. proposed a technical model for the first time. Aiming at the problem of repeated payment of existing third-party electronic payment, this paper attempts to establish a decentralized point-to-point direct transaction e-cash system based on P2P network technology and uses cryptography, timestamp, and consensus mechanism to ensure that the data is easy to trace but not easy to tamper [21]. Zhu and Zhou analyzed the security and performance of different consensus mechanisms and network parameters in public by introducing a new quantitative framework [22]. The framework can objectively analyze the trade-off between the performance and security of public blockchain under the conditions of network propagation, block size, block generation interval, information propagation mechanism, and network malicious attack. Nathan and Jacobs proposed a private blockchain evaluation framework [23]. At present, the design of BLOCKBENCH is carried out through three most mature blockchain platforms that can support smart contract functions: Hyperledger Fabric, ETHEREUM, and PARITY. Cong and He put forward the model of Ethereum and developed a programming script language with complete Turing and more complex intelligent contracts for the first time based on technology [24]. delegated Byzantine fault tolerance (DBFT) is an improved Byzantine fault tolerance algorithm initiated by domestic small ant team, which has been applied to its original ant shares (NEO) project [25].

Therefore, through a unified input-output interface, the underlying code of performance simulation evaluation of consensus algorithm is integrated into the platform visualization management module, which is convenient for blockchain practitioners to start performance simulation by inputting certain specific parameters.

## 3. Combination of Financial Efficiency and Supervision and Structural Optimization Method

### 3.1. Combination Method of Financial Efficiency and Financial Supervision

**Figure 1:**
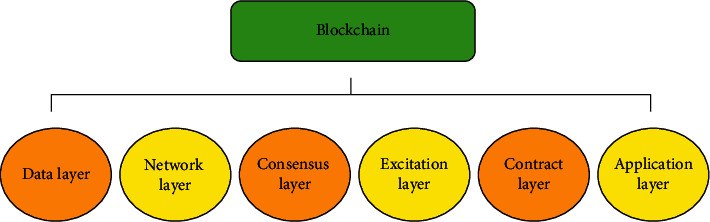
Blockchain infrastructure model.

In the current credit economy, financial resources are fresh resources required to maintain social and economic progress [26]. The Pareto optimality criterion is extended and applied to resource allocation [27]. The Pareto optimal allocation of financial resources is then the so-called financial efficiency. As a result, advancements in financial technology not only raise the risk of consumer information disclosure and fraud, but also make customer personal information protection and remedy more difficult. These are regulatory concerns that need to be addressed as financial technology develops. Data layer, network layer, consensus layer, incentive layer, contract layer, and application layer are the six levels that the blockchain system separates its infrastructure into, from low to high, according to distinct functions. The blockchain infrastructure model is shown in Figure 1.

Firstly, according to the Pareto condition of optimal distribution of commodities among consumers, when there are two or more consumers and two or more commodities, when the marginal substitution rate between two commodities is the same for any one consumer, and when it is equal to the ratio of product price, the distribution of commodities will reach Pareto optimal. Financial regulators are responsible for supervision. Select the appropriate key generation function according to the different encryption methods of each part, which must meet the conditions under the following formula:

(1) $$\begin{array}{c} K_{2i}=f_{i}\left(U_{i}R_{i},H_{i}\left(CF\right)\right). \end{array}$$

The blockchain is distributed and stored at each node of the network in the form of encryption. All transaction records of each node of the whole network are stored on the chain, so the blockchain can be regarded as an account book recording all bitcoin transactions [28]. The cost of regulators' hard work will be significantly higher than the cost of laziness, while the benefits of hard supervision and laziness are not obvious. Therefore, the net benefits of regulators' hard work will be significantly lower than the net benefits of laziness, that is,

(2) $$\begin{array}{c} \left(R_{2}-C_{2}\right)>\left(R_{i}-C_{i}\right),\left(R_{2}^{*},C_{2}^{*}\right)>\left(R_{1}^{*},C_{1}^{*}\right). \end{array}$$

Blockchain technology has a natural trust advantage, because it cannot be tampered with, is easy to trace, and is very suitable for scenarios where some information is opaque, credit risk is high, and losses may be high [29]. Set up various financial supervision departments corresponding to different types of financial institutions and delimit the jurisdiction of each financial supervision department, and the supervision departments of one type of financial institutions shall not supervise other types of financial institutions beyond their authority. At this time, there is also a refined Bayesian Nash equilibrium, where regulators choose to be lazy, and the public choose to pay low remuneration when

(3) $$\begin{array}{c} p=\frac{\left(R_{p}^{*}-C_{p}^{*}\right)-\left(R_{p}^{*}-C_{p}^{*}\right)}{\left(R_{\text{q}}-C_{q}\right)}. \end{array}$$

Secondly, according to the Pareto condition of the optimal allocation of producer input factors, the input combination of the optimal allocation of producer input factors is an input combination that meets the equal marginal rate of technology substitution of any two inputs and is equal to the ratio of input factor prices. Through multiple transaction intermediaries, the transaction efficiency is greatly reduced, the transaction cost is increased, and the moral hazard is high. The application of blockchain technology can realize point-to-point direct transactions with high trust of all trading parties, so as to improve efficiency and reduce costs. Use performance indicators to express the expected objectives of the system:

(4) $$\begin{array}{c} J=f_{t0}^{tf}L\left[x\left(t\right),u\left(t\right),t\right]\text{d}t. \end{array}$$

Then, combined with the encryption algorithm, the transaction results are made public (that is, the prototype of blockchain), and a decentralized property authentication system is established. Due to the specialized operation of the supervised subjects, the supervision level is highly specialized, which greatly guarantees the stability and security of the financial system in a certain period of time. The method of selecting the optimal process and strategy can be solved by establishing Hamiltonian function:

(5) $$\begin{array}{c} H\left(x,u,\lambda ,t\right)=L\left(x,u,t\right)+\lambda \left(t\right)f\left(x,u,t\right). \end{array}$$

Finally, for financial resources, if the supply and demand of financial resources are to be roughly balanced, that is, to meet the distribution of financial resources among financiers and financial commodities among investors, the marginal financing conversion rate of financiers must be equal to the marginal commodity substitution rate of investors. If the business scope of financial institutions is clearly and strictly divided, and the responsibility of financial supervision is relatively clear, the separate supervision system of institutional supervision is feasible. Since financial institutions only need to accept the supervision of one regulatory department, they can avoid the cross supervision and overlapping supervision of regulatory departments, reduce the cost of regulators, and indirectly reduce the cost of consumers. Any authorized person can see the records of the whole data chain at any node, so that the audit department and regulatory department will celebrate warmly.

### 3.2. Optimization Method of Financial Supervision Structure

Vulnerability is the fundamental motivation of financial supervision and the core and springboard of long-term historical development of financial supervision [30]. With the increasingly blurred division of business among heterogeneous financial institutions, institutional supervision is likely to lead to gaps in financial supervision and potential risks, especially the subsidiaries controlled by financial groups can carry out banking, securities, and insurance business separately. Therefore, it is necessary to realize the stability of monetary function and financial function, including the stability of financial market function and the stability of financial enterprises. The optimal control problem is defined by mathematical language; that is, the state equation of the given continuous-time economic system is

(6) $$\begin{array}{c} x^{\circ }\left(t\right)=f\left[x\left(t\right),u\left(t\right),t\right]. \end{array}$$

**Figure 2:**
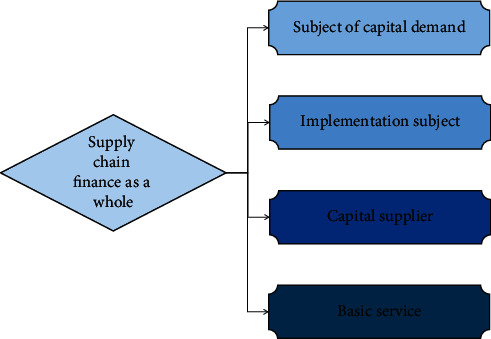
Overall financial application structure model of supply chain.

That is, under the background of computer-driven trading, the trading frequency and trading volume are rising rapidly. Therefore, the structure of financial supervision needs to be optimized. On the basis of giving full play to the role of basic services, the party applying funds, the party providing funds, and other participants in the supply chain are constructed together, as shown in Figure 2.

First, handle the relationship between financial supervision and financial supervision structure adjustment. Under the condition of modern financial economy, the investment of financial resources comes from savers' savings in a broad sense, including the financial assets investment behavior of individuals and enterprises. When implementing the institutional supervision mode, even if the main supervision system is adopted for the business approval and supervision of a certain type of financial institutions, and several financial supervision institutions are responsible for coordination, the supervision objectives are diverse, not a single one. The real economic system, in addition to its constantly changing environment, will also change its internal structure, thus affecting the performance of the economic system. The general equation of adaptive control is

(7) $$\begin{array}{c} \text{A}\left(Z^{-1}\right)Y\left(t\right)=Z^{-d}B\left(Z^{-1}\right)u\left(t\right)+C\left(Z^{-1}\right)W\left(t\right). \end{array}$$

Before extracting the main features of distributed big data from the database, set the evaluation criteria of distributed data features, and apply them when evaluating the importance of features to determine the ability to distinguish feature classes. When publishing data on the blockchain, everyone can access it and issue transactions waiting to be written into the blockchain. Suppose that *S*=*S*(*i*) represents the surplus of consumers or investors of financial products, and Π=*π*(*i*) represents the profit of financial enterprises. They are all functions of financial market price. Then, the political production function is as follows:

(8) $$\begin{array}{c} M=M\left(S,\pi \right). \end{array}$$

Secondly, the compatibility between regulatory structure and regulatory objectives: the setting of regulatory structure should ensure the smooth realization of regulatory objectives and reduce the interference of structure to regulatory objectives. This condition shows that the marginal substitution rate between any two commodities is equal to the marginal product conversion rate of producers between these two commodities. Therefore, a possible boundary of political production can be deduced:

(9) $$\begin{array}{c} S=S\left(i\left(\Pi \right)\right)=S\left[\pi^{-1}\left(\Pi \right)\right]. \end{array}$$

Since one of the purposes of blockchain is to reduce excessive human interference, it emphasizes automation. Through the setting of mathematical algorithms and the formulation of some rules, transactions should be automated. We should not only maximize the number of votes, but also make the marginal substitution rate of politics equal to the marginal substitution rate of mutual transfer between enterprise profits and consumer surpluses, so as to achieve a balance. Therefore, the interest rate of financial supervision is solved as follows:

(10) $$\begin{array}{c} {\text{max}}_{i}M\left(S\left(i\right),\left(i\right)\right). \end{array}$$

**Figure 3:**
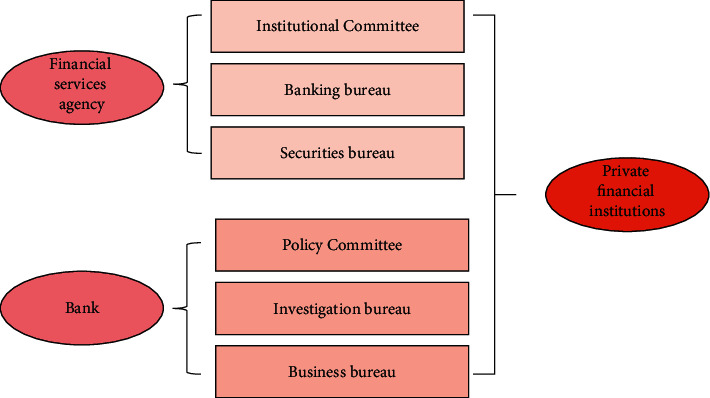
Financial supervision structure model.

Less than 51% of the nodes in the system can intervene and verify the transaction; otherwise, a single node cannot play any role in data tampering, and at the same time, the nodes of the whole network will be alert to it, and it will be automatically rejected by the system. The financial supervision structure model is shown in Figure 3.

Finally, the financial supervision system must be based on market law and cannot substitute subjective administrative intent for the market mechanism. Its goal should be to support financial innovation, financial efficiency, and financial stability. Anyone may find the information they need thanks to binary value (hash value) query entries, which make the system's data information more transparent. The use of blockchain in bitcoin is to use the blockchain to validate blocks containing transaction data in order to achieve the goal of irreversible and undisputed transaction confirmation. Each regulatory authority's jurisdiction is determined by the kind of regulated financial institutions such as commercial banks, securities firms, and insurance firms. The regulatory structure optimization is based on cryptographic algorithm technology, which ensures the unique direction of account address and has some application potential in the areas of authentication and company identity certificate.

## 4. Blockchain and Consensus Algorithm Analysis

### 4.1. Analysis of Blockchain Core Technology

When the blockchain was first proposed, it was designed to solve the problem of safe transaction between the two parties in the unknown network environment without a third-party certification authority, that is, to create a payment mechanism through communication channels without a trusted party. Therefore, only by using blockchain to supervise financial institutions can the government overcome the negative impact of market failure, improve the governance level of financial institutions, improve the efficiency of financial operation, and maintain the stability of the financial system.

Firstly, distributed ledger refers to the accounting technology of data sharing, data replication, and data synchronization among nodes in computer network. Pow, namely, workload proof, is a consensus mechanism, which first appeared in bitcoin. An input value of any length can be calculated into a binary value of a specified length. For example, the SHA-256 algorithm applied in the blockchain system can calculate the fixed length output with the growth of 256 bits for transactions or other data of any length, and the output binary value is hash value (also known as hash value). Learn from the data processing mechanism of separate storage of cold and hot data and separate storage of cold and hot data in traditional financial data processing methods to realize the effective storage of a large number of node data. When a node reaches the target value, it will broadcast the block to other nodes, and all other nodes must confirm the correctness of the hash value with each other. The specific transaction information is stored in blocks, and the data structure of blocks is shown in Table 1.

Secondly, in the blockchain system, all transaction data is publicly visible, but the personal identity information of the nodes is encrypted, and only after the information owner authorizes the nodes to access the information, which is to protect the security of transaction data and the privacy of personal identity information. The data structure of the header is shown in Table 2.

Blockchain system is a point-to-point network composed of a large number of nodes. There is no centralized hardware or management organization. The whole system depends on the cooperation of all nodes in the network, and all nodes have the same business mode, rights, and obligations. POW collects all pending transactions on the network after creating the last block. Then, the transaction data received within a certain period of time forms a Merkle tree data structure through hash operation at each distributed node, encapsulates the nodes that have obtained the consensus mechanism, connects the current longest main chain, and creates the nearest main chain. The Merkle root of these transactions is then calculated and filled with block serial number, 256-bit hash of the previous block, current target hash, random number, and other information. In order to release data uniformly and avoid problems between the two armies, some specific data processing links can only be processed in serial mode, not in parallel.

Finally, the consensus mechanism is a method to prove the correctness and legitimacy of a block by reaching consensus among all the nodes participating in the consensus in the blockchain network environment. Since there is only one “Merkel root” at the end of processing a large amount of transaction data, it is not necessary to encapsulate all the underlying data in the block header, which greatly improves the operation efficiency and scalability of the blockchain. The consensus mechanism depends on the ability of the kernel, using CPU and memory resources to isolate the operating system's view of applications through a separate namespace.

### 4.2. Consensus Algorithm Analysis

**Figure 4:**
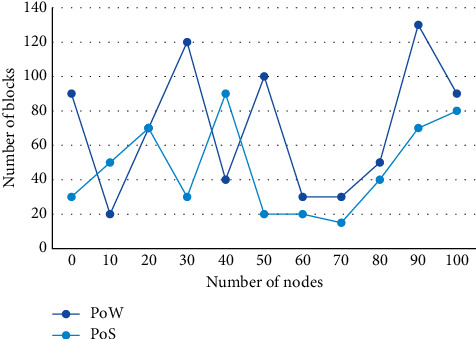
Comparison diagram of block broken lines generated by PoW and PoS within 100 minutes.

**Figure 5:**
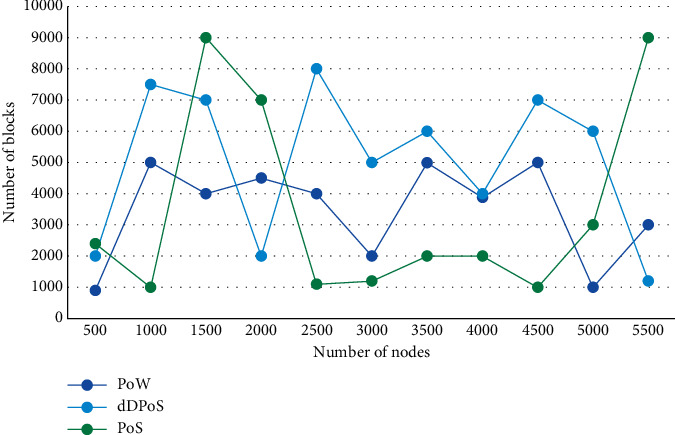
Comparison diagram of block broken lines generated by PoW, PoS, and dDPoS under 5000 nodes.

POW competes for the computing power of each node and then competes for the power of generating blocks, which greatly consumes computer resources and power resources. The submodule will regularly poll the status of the sent transaction request until all the unconfirmed transactions in the local sequence are confirmed and removed from the sequence, and all the transaction requests sent by the client are completed. The network environment composed of consensus nodes is called consensus network. In the dDPoS algorithm, the consensus network changes with the change of consensus nodes. In addition, the consensus efficiency of pow is too slow. It takes 10 minutes to generate a block. Among them, the web module provides basic web-oriented integration features. The web servlet module package contains the spring MVC implementation of web applications. The number of consensus nodes elected by DPoS consensus algorithm is 100, which has the advantages of reducing the number of consensus nodes, improving consensus speed, and reducing energy consumption in the consensus process. This indicator represents the transaction response time of the blockchain and can directly reflect the performance of the consistency algorithm. The number of blocks generated by the three consensus algorithms pow, POS, and dDPoS in 0–100 minutes and under nodes with different sizes of 500–5500 is shown in a broken line diagram, as shown in Figures 4 and 5.

**Figure 6:**
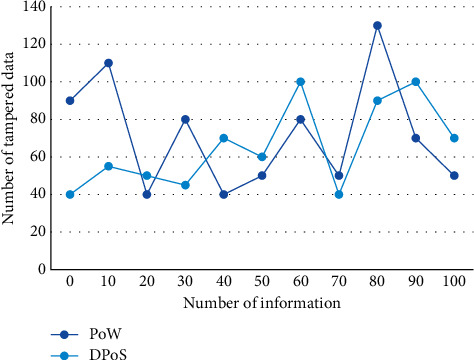
Comparison line chart of average delay of consensus algorithm under different transaction requests.

Firstly, select the consensus node module: functionalize all nodes in the blockchain system, and different types of nodes undertake different tasks, which are mainly divided into consensus nodes and transaction nodes. During the change of transaction request rate from 20 times per second to 200 times per second, the average delay of PoW first increased and then decreased, while the delay of DPoS fluctuated around 5 seconds, during which the peak delay of PoW reached 50 times that of DPoS. The logarithmic graph of average delay of PoW and DPoS consensus algorithms at different transaction request rates is shown in Figure 6.

**Figure 7:**
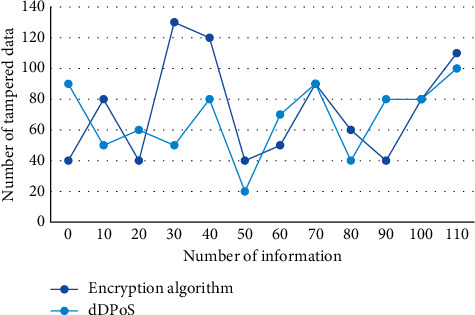
Comparison of antiattack performance between encryption algorithm and dDPoS algorithm.

The blocking efficiency of the dDPoS consensus algorithm is about one block every 10 seconds, which is much higher than that of the pow consensus algorithm. Data entry permission can set multiple users in a unified account and provide users with different work permissions according to their different work needs, so as to meet the use scenarios under the control of multiple users. All data are extracted from the financial database of decentralized supply chain and mapped into a multidimensional space. This is because some interest groups may not make public support or opposition after cost-benefit comparison. On the one hand, the demand for financial supervision system depends on the comparison between the number of beneficiary groups *n* and the number of damaged groups (*N* − *n*); on the other hand, it depends on the degree of support *f* expressed by beneficiary groups and the degree of opposition *h* expressed by damaged groups. The security and integrity of information and data in the decentralized supply chain financial network can be better protected.  Figure 7 shows the antiattack performance comparison of encryption algorithm and dDPoS algorithm.

**Figure 8:**
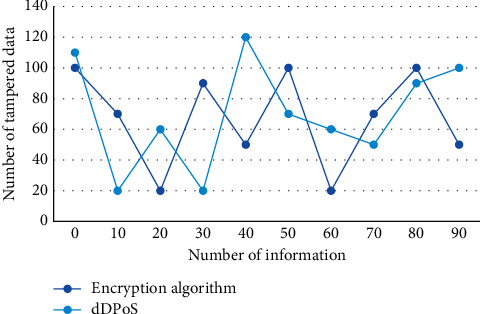
Comparison of protocol security in distributed network.

Secondly, remove the malicious node module: when the malicious node appears, dDPoS algorithm enters the malicious node removal mode and introduces the upgrade mechanism to complete the upgrade conversion between the malicious node and the candidate node. During data processing, ECDG, PCI, and ＲDTP protocols were tested, and the final test results are shown in Figure 8.

Filter much transaction information of blockchain finance through artificial intelligence, big data, and cloud computing technology, and write algorithms and models that can automatically calculate and analyze these data to capture, identify, and analyze abnormal behaviors of regulatory objects. One of the problems to be solved in the initial design of blockchain system is the problem of repeated payment in electronic transactions; that is, a sum of money cannot appear in two transactions. It is a method of filtering data features using split equation and a feature extraction method for calculating the score corresponding to size features. In particular, it should be noted that some damaged groups may evade regulation, which mainly depends on the comparison between the net income of publicly opposing the success of regulation and the net income of evading regulation.

## 5. Conclusions

The rapid advancement of financial technology may provide significant benefits to regulators. Blockchain technology, as opposed to the old centralized approach, uses cryptography, distributed consensus, and economic incentives to achieve the benefits of decentralization, nontampering, data traceability, and programmable smart contracts. We can create a blockchain-based evidence retention and collection system based on this characteristic of blockchain technology. Financial supervision not only necessitates a genuine increase in the enthusiasm for nongovernmental financial industry oversight, but also actively improves the independence of financial supervision organizations. The goal of effective supervision is to reduce the expense of supervision while increasing the contribution to social welfare.

This study proposes a strategy for integrating financial efficiency and oversight, as well as a method for structural optimization, based on blockchain. In addition, the blockchain's underlying technology and consistency algorithm are thoroughly examined. This paper effectively integrates relevant theories and knowledge of financial supervision and financial efficiency, with the goal of improving financial efficiency. It attempts to reveal the mechanism of financial supervision on financial efficiency and discusses the impact of financial supervision on improving financial efficiency. It can not only assist blockchain practitioners understand the characteristics of consistency algorithm and make acceptable decisions, but it can also improve the performance simulation efficiency of consistency algorithm and promote further research into consistency algorithm. It can help related businesses develop and process information more quickly, allowing for the effective and practical application of blockchain technology. It can also help solve problems with the original model, allowing the new model to be implemented and popularized more quickly, contributing to the development of a good trade settlement environment in China. This can help financial organizations fix their financial transaction data, prevent tampering and data loss, and preserve the integrity of their financial data.

## Figures and Tables

**Table 1:** Block data structure.

Field	Describe	Size (byte)
Block size	The size of all information after this field	1–10
Block head	The general name of all the information constituting the block header	92
Number of transactions	The number of transaction information stored in the block	1–20

**Table 2:** Data structure of block header.

Field	Resource consumption	Size (byte)
Pervious block hash	Enormous	20
Merkle tree root	Big	87
Timestamp	Small	20–30

## Data Availability

The data used to support the findings of this study are available from the corresponding author upon request.
